# Cardiorespiratory Fitness in Volleyball Athletes Following a COVID-19 Infection: A Cross-Sectional Study

**DOI:** 10.3390/ijerph18084059

**Published:** 2021-04-12

**Authors:** Aleksandra Milovancev, Jovana Avakumovic, Nemanja Lakicevic, Valdemar Stajer, Darinka Korovljev, Nikola Todorovic, Antonino Bianco, Nebojsa Maksimovic, Sergej Ostojic, Patrik Drid

**Affiliations:** 1 Faculty of Medicine, University of Novi Sad, 21000 Novi Sad, Serbia; aleksandra.milovancev@mf.uns.ac.rs; 2 Health Center “Novi Sad”, 21000 Novi Sad, Serbia; avakumovic.jovana@dzns.rs; 3 Sport and Exercise Sciences Research Unit, University of Palermo, 90133 Palermo, Italy; lakinem89@gmail.com (N.L.); antonino.bianco@unipa.it (A.B.); 4 Faculty of Sport and Physical Education, University of Novi Sad, 21000 Novi Sad, Serbia; stajervaldemar@yahoo.com (V.S.); korovljev.darinka@gmail.com (D.K.); nikolatodorovic1708@gmail.com (N.T.); nebojsam@uns.ac.rs (N.M.); sergej.ostojic@chess.edu.rs (S.O.)

**Keywords:** cardiorespiratory fitness, spirometry, performance, volleyball, detraining, respiratory muscle training

## Abstract

Athletes’ lifestyles have been dramatically affected by the coronavirus disease 2019 (COVID-19) pandemic. Since COVID-19 primarily affects the respiratory system and to a lesser degree the cardiovascular system, the goal of this study was to examine the effects of COVID-19-caused detraining on cardiorespiratory fitness (CRF) of recently recovered volleyball athletes. Sixteen experienced volleyball athletes (age 24 ± 4.5 years) who were recently diagnosed and recovered from a COVID-19 infection volunteered to participate in this study and were tested for CRF and spirometry. Given that participants had only mild symptoms of infection, the primary focus of this study was on the effects of detraining on CRF. On average, the time to exhaustion was 9.4 ± 1.4 min. VE, VCO_2_, RER and oxygen pulse increased, heart rate exceeded 90% of predicted values, and peak VO_2_ values were typical for this level of athlete (44.1 ± 3.4 mL/kg). Pulmonary function reflected in FVC, FEV1/FVC and MVV values were well above 80% of predicted values for each of the participants while electrocardiography revealed no ischemia, arrythmias or conduction and repolarization abnormalities were found in the tested subjects. Therefore, it can be concluded that participants experienced typical consequences of detraining. Due to a lack of CRF data prior to COVID-19 infection, we were unable to estimate the magnitude detraining had on CRF. Complete CRF assessment after COVID-19 infection in athletes can be useful for screening of residual myocardial and/or respiratory system damage for safe return-to-play decisions.

## 1. Introduction

Ever since the coronavirus disease 2019 (COVID-19) outbreak emerged at the end of 2019 in a multimillion city in central China, patterns of our daily life have been dramatically changed [1]. In the absence of an effective medication or vaccine, the World Health Organization and various governments around the world have emphasized personal efforts such as temporary lockdown, social distancing, face mask wearing, increased hand hygiene, etc., to minimalize the risk of viral spread [2]. As vaccines and other non-pharmacological aids are being applied on a global scale, COVID-19 rules are still in place to a smaller or larger extent depending on distinct laws established by individual countries. Athletes have not been exempted from the abovementioned rules and thus the majority of competitions have been cancelled, which has had a profound impact on athletic life [3]. Nevertheless, many athletes were unable to proceed with their usual training routines [4] due to prohibitions imposed by the law, and this pertains even more so to athletes who partake in team sports, especially those that are played indoors [5]. This pattern of capriciously dispersed training sessions may disrupt normal training periodization, which is an essential part of the training design [6], and can consequently impair performance and competition status once the competitions are allowed. For a majority of athletes, this has led to a reduced level of physical activity, which might be a blessing in disguise, since studies have repeatedly shown that heavy exercise often practiced by athletes can have a temporary immunosuppressive effect [7,8], which can likely increase the susceptibility to COVID-19 infection. However, moderate-intensity physical activity has beneficial effects on the immune system [9,10] and it is crucial for individuals to stay active during the COVID-19 pandemic [11,12] as moderate doses of exercise on select immune markers are associated with many disease states, and can be immuno-protective in patients who contract SARS-CoV-2 [13].

To make matters worse for athletes, some of them have contracted the COVID-19 infection and this occurrence can certainly have negative effects both on their heath and career trajectories. Since COVID-19 infection primarily affects the respiratory system and to a smaller degree the cardiovascular system [14], the aim of this study was to examine the effects of COVID-19-caused detraining on the cardiorespiratory status of volleyball athletes briefly after infection.

## 2. Materials and Methods

### 2.1. Participants

The sample consisted of sixteen male Serbian first division volleyball players (24 ± 4.5 years; 193.4 ± 9.9 cm; 90 ± 8.9 kg) who volunteered to participate in this study (Table 1). All participants had a recent COVID-19 infection and have fully recovered and returned to their everyday sports activities prior to testing in an exercise physiology laboratory (Table 2). Once they were verbally familiarized with the testing protocol, participants gave their written informed consent to participate in the study after receiving a thorough explanation of the study protocol. The Ethical Committee approved the study of the Faculty of Sport and Physical Education (Ref. No. 46-06-02/2020-1), University of Novi Sad, and the experiment was conducted according to the Helsinki Declaration’s principles.

### 2.2. Pre-Testing Procedure

To determine the severity of COVID-19 and to avoid possible side effects during testing, we asked a series of questions regarding the symptoms participants suffered during the infection. Hereby, we addressed commonly reported symptoms such as temperature, dry cough, fatigue, headache, etc., which were then combined with a Physical Activity Readiness Questionnaire (PAR-Q) to gather comprehensive retrospective data on involved participants. Participants were excluded if they had any significant musculoskeletal injury, blood pressure over 150/90 mmHg (OMRON M7, OMRON, Kyoto, Japan.) or any abnormalities in a pre-check electrocardiography (E30G FARUM S.A., Poland). Participants had no residual symptoms of COVID-19 in the moment of CRF testing.

### 2.3. Testing

All athletes had a single visit to the lab, and evaluations were performed in the same period of the day. They were instructed to avoid strenuous activities 24 h before the evaluations. Initially, body height was determined using a portable stadiometer SECA 213 (SECA Inc.-Hamburg, Germany) while body mass and body composition components percentage of fat and muscle mass were determined using Omron BF511 bioelectric impedance analysis (Omron Inc. Osaka, Japan).

Pulmonary function and ECG were conducted by a medical doctor. Forced vital capacity (FVC), Forced expiratory volume in 1 s (FEV1), FEV1 as a percentage of FVC (FEV1/FVC), and maximal voluntary ventilation (MVV) values were obtained via Spirolab II (MIR Inc. Rome, Italy). All athletes were evaluated before cardiopulmonary exercise testing (CPET), using a standard 12-leads ECG Unit-E30G (Farum, S.A., Warsaw, Poland), with standard calibration 10 mm, equal to 1 mV and 25 mm/s paper speed. With this initial approval, athletes were able to continue CPET.

Cardiorespiratory fitness was determined by a running-to-exhaustion incremental test on a Cosmed T170 treadmill (COSMED, Rome, Italy) (1 min standing still, 3-min warm-up walk at 6 km/h followed by running at 8 km/h with progressive workload increment rate of 1.5 km/h every 90 s until exhaustion and a period of three minutes of recovery). Cardiorespiratory data were collected using a breath-by-breath metabolic system Quark CPET (COSMED, Rome, Italy) and a heart rate monitor Polar RS800cx (Polar, Oy Kempele, Finland). Evaluated variables were: Work rate in watts—WE (W)—; Respiratory ventilation—VE; Oxygen uptake milliliters per kilogram per minute—VO2; Carbon dioxide production liters per minute—VCO2 (L/min); Respiratory exchange ratio—RER; Ventilatory equivalent for carbon dioxide production VE/VCO2; Oxygen uptake milliliters per heartbeat—O2 pulse; Heart Rate beats per minute—HR; Ventilatory equivalent for oxygen uptake—VE/VO2. The conducted test variables were presented in the state of rest, exhaustion peak values, for the first and second ventilator threshold, and each of the three minutes during the recovery phase.

### 2.4. Statistical Analysis

The descriptive statistics were expressed for each variable using the SPSS statistical package (version 24 for Windows, Chicago, IL, USA). To better identify the possible effects of COVID-19 on CPET results and to provide a comparative perspective, we conducted an independent *t*-test analysis (MedCalc Software Ltd., Ostend, Belgium) comparing our results to a similar cohort in different studies.

## 3. Results

Sixteen participants had their COVID-19 infection confirmed via a Polymerase Chain Reaction (PCR) test. Twelve participants reported having above-average temperature (37 ± 0.8), while ten reported having fatigue and a loss of smell and taste during the infection (Table 2). None of the participants were hospitalized, nor were they taking any medications while infected. Instead, their physician referred them to stay at home and boost their immune system, since they had only mild symptoms. Therefore, the severity of illness experienced participants suffered can be classified as mild. On average, they had symptoms of the virus for one week. Nevertheless, two weeks of quarantine was mandatory. Therefore, participants were unable to train for a total of 22 days. Participants reported achieving more significant volumes of training after quarantine (18.5 ± 6.2 h/week) compared to the period prior to infection (16.2 ± 6.2). This phenomenon can be seen as the athletes’ effort to compensate for endurance and strength gains lost during the period of infection, which inevitably led to detraining. However, no details about training frequency or intensity were acquired.

The entire cohort has completed the testing protocol (Table 3). The time to exhaustion was 9.4 ± 1.4 min, which can be classified as normal values for running-to-exhaustion incremental tests. During the testing procedure, VE, VCO_2_, RER and oxygen pulse increased, heart rate exceeded 90% of predicted values, and peak VO_2_ values were typical for this level of athlete (44.1 ± 3.4 mL/kg) [15].

Interestingly, both first and second ventilatory thresholds in our study (VT1 = 73% and VT2 = 92.5%, respectively) were above-average values (Table 3). VT1 and VT2 are indicators that should be determined to help exercise prescription in cardiac disease concerning exercise safety and efficiency. Normally, it varies between 40–60% of the VO_2peak_ for VT1 and 60–90% for VT2 for a healthy population. Following the completion of CPET, all variables were followed up during the first three minutes of recovery, and no abnormalities were noted as athletes reached the steady-state level. Concerning pulmonary function, FVC, FEV1/FVC and MVV values were well above 80% of predicted values (FVC_(L)_ = 5.3 ± 2.2; FEV-1 _(L)_ = 4.7 ± 1.9; FEV1/FVC _(%)_ = 90.5 ± 8.2; MVV = 147.7 ± 64.8 _(L/min)_) for each of the participants. ECG testing revealed no ischemia, arrythmias or conduction and repolarization abnormalities in the tested subjects.

## 4. Discussion

The aim of this study was to examine the effects of COVID-19-caused detraining on cardiorespiratory function in volleyball athletes. Since participants experienced only mild symptoms of COVID-19 infection, the focus of this study was not on infection per se but rather on the detraining period that was inevitably associated with the infection. It should be noted that participants involved in this study had a period of approximately 20 days of training prior to engaging in CPET, which leaves a short leeway for athletes to possibly alleviate consequences of detraining and perhaps return to their pre-infection values. Therefore, obtained CPET data can be classified as standard for their age and level of competition. In addition, FVC, FEV1/FVC and MVV values were well above 80%, indicating relatively normal pulmonary function while ECG testing revealed no cardiac abnormalities in the tested subjects.

Similar studies examining CPET in volleyball players have been conducted previously [16,17,18]. It is important to note that subjects from other studies were healthy and regularly trained volleyball players and did not experience a COVID-19 infection. The analyzed results show that subjects within our study had lower VO_2peak_ and VT2 when compared to healthy and training-undisturbed professional and amateur counterparts [16,17,18]. Only in one study [17] on college volleyball players was there no statistical difference in VT2, since these subjects were college athletes. For practical purposes, comparing speed at peak and VT2 can be more relevant to practitioners to see apparent differences in athletes’ running economy. When comparing results for peak values, we can see that athletes within the present study had better or similar results compared to these studies [16,17]. An even better discriminator of difference between national and elite level can be VT2 speed. A similar conclusion was seen in the study by Djurkovic [16].

In the context of the COVID-19 aftermath, somewhat similar findings were found in a study by Clavario et al., [19] where 34.5% of the COVID-19-affected participants (non-athletes) had below VO_2max_ values, whereas 65.5% had above the 85% predicted value, which is indicative of normal values. Furthermore, pulmonary function test parameters were within the normal limits. Authors concluded that a functional capacity limitation found in some participants can be mainly explained by muscular impairment, while cardiopulmonary causes should not be excluded. It should be pointed out that the abovementioned study included lay people and not exclusively athletes like we did. Furthermore, included samples were tested three months after hospital discharge, whereas participants in our study had around three weeks of detraining and roughly three weeks of re-training. However, in a study by Raman et al., [20] VO_2max_ and ventilatory efficiency on CPET and six-minute walk distance (405 ± 118 m vs. 517 ± 106 m in controls, *p* < 0.0001) were significantly reduced in COVID-19 survivors 2 to 3 months after the infection ended. Moreover, the degree of extra-pulmonary magnetic resonance imaging-detected abnormalities and exercise tolerance correlated with serum markers of ongoing inflammation and severity of acute illness.

A major limitation of this study was the lack of data with respect to frequency and intensity of training performed by the investigated athletes for a period of approximately 3 weeks after the detraining period, but before data collection. This time-frame gave athletes the opportunity to partially mitigate the effects of detraining and contributed to recovery leading to pre-infection values. Certainly, knowledge of CPET and spirometry values of tested subjects prior to infection would have allowed us to determine the magnitude that infection and subsequent detraining had on tested variables. Another limitation is the lack of control group whereby non-infected volleyball athletes of similar training status and anthropometric characteristics would be compared to their infected counterparts. However, as athletes around the world are coming back to their usual training routines and competitions, our study addressed the topic that is very current and pertains to many athletes who were infected with COVID-19. Moreover, our study may be an indicator for developing a post-COVID-19 screening protocol that athletes ought to undergo prior to returning to their training and competition.

As a focal point of this study, detraining is defined as a partial or complete loss of training-induced adaptations [21] which, for our cause, pertains mainly to endurance training. Although in this case it is relatively short, detraining can have a significant impact on the previously gained benefits of endurance training. In a study by Coyle et al., [22] it has been shown that as little as 12 days can markedly reduce VO_2max_ solely due to decreased stroke volume as heart rate and arteriovenal difference stayed the same during this time frame. This quick decrease in stroke volume seems to be due entirely to a rapid loss in plasma as endurance training is ceased. Moreover, after approximately three weeks of detraining (which is the case in our study), a drop in arteriovenal difference is noted, which is associated with a reduction in muscle mitochondria while capillary density remains stable [23]. Two studies showed that detraining lasting 42 to 85 days can significantly reduce the oxidative capacity of skeletal muscle and even decrease the percentage of type IIa (intermediate) muscle fibers from 43% to 26% and increase the percentage of type IIx muscle fibers from 5 to 19% [22,23]. Consequently, studies have shown that during short-term detraining there is an increased reliance on carbohydrate as a primary substrate for energy production during exercise, at both submaximal [23,24,25] and maximal intensities [26]. This pattern has also been detected in our study as RER values were reaching 1.19 at peak HR values. A higher reliance on carbohydrate as the primary fuel during exercise might be partially explained by a decrease in muscle lipoprotein lipase activity during detraining [27], but is more likely due to significantly reduced sensitivity of insulin-mediated whole-body glucose uptake associated with short-term detraining [28,29,30]. Interestingly, detraining of two months had similar cardiac effects in both younger and older athletes with wall thicknesses decreased only in young athletes, while left ventricular mass and end-diastolic diameter and volume was reduced only in older athletes [31]. These findings imply that younger age does not necessarily offer protective properties to detraining, at least in terms of cardiology. Moreover, both younger and older individuals experience benefits of a similar magnitude with regular endurance training [32]. The overall incidence of COVID-19-related cardiorespiratory issues, especially myocardial injury in competitive athletes and the impact on sports participation, remains undetermined. Early reports revealed post-recovery heart damage in 78% of COVID-19 patients [33], while Rajpal et al. [34] reported 15% of competitive athletes had myocarditis, which can be a major cause of sudden cardiac death in athletes. However, in our case, yielded ECG data showed regular cardiac patterns with no noticeable abnormalities.

## 5. Conclusions

Even though participants in this study have showed optimal CPET results, we highly recommend that in addition to medical clearance, athletes should perform CPET and spirometry prior to returning to their training regimen as the literature shows consistent reports that COVID-19-positive athletes may experience persistent and residual symptoms up to several months after initial infection, reflected in a cough, tachycardia and extreme fatigue. Therefore, complete CRF assessment after COVID-19 infection in athletes can be useful for screening of residual myocardial and/or respiratory system damage for safe return-to-play decisions.

## Figures and Tables

**Table 1:** Sample characteristics.

Age _(years)_	24 ± 4.5
Height _(cm)_	193.4 ± 9.9
Weight _(kg)_	90 ± 8.9
BMI _(kg/m^2^)_	24.3 ± 2.4
FM_%_	17.6 ± 3.4
MM_%_	40.3 ± 2.2
SBPc/DBPc	129 ± 12.7/74 ± 12
Sport experience _(years)_	11.4 ± 6.4
Pre COVID Training for week _(h)_	16.2 ± 5.5
After COVID Training per week _(h)_	18.5 ± 6.2
Training cessation _(days)_	22 ± 7
Training prior to testing _(days)_	20.1 ± 4.7

Legend—Abbreviation: BMI—Body mass index; BF_%_—Body fat precent; MM_%_—Muscle Mass percent; SBPc—Systolic blood pressure-(mmHg); DBPc—Diastolic blood pressure-(mmHg).

**Table 2:** COVID-19 symptoms.

	*N*	Mean ± SD
Temperature	12 (75%)	37.7 ± 0.8 °C
Dry Cough	5 (31%)	
Fatigue	10 (62.5%)	
Muscular Pain	8 (50%)	
Chest Pain	1 (6%)	
Headache	5 (31%)	
Loss of Smell and Taste	10 (62.5%)	
Diarrhea	3 (19%)	
Difficulty in Breathing or Shortness of Breath	1 (6%)	
Lost Weight	1 (6%)	2.5 kg
Symptoms in days	16	7 ± 6.8
Symptoms confirmed by PCR test	16	

**Table 3:** Characteristics of—CPET.

				Time to Exhaustion: 9.4 ± 1.4 min			
	Rest	Peak	VT2	VT1	Reco1	Reco2	Reco3
WR _(W)_	0	173.3 ± 27.8	111.3 ± 26.5	74.9 ± 14.8	74	74	74
VE _(L)_	21 ± 4.1	152.4 ± 18.7	108.6 ± 12.3	72.8 ± 11.0	110.5 ± 18.5	82 ± 13.7	68.1 ± 12.1
VO_2 (mL/min/kg)_	6.7 ± 1.0	44.1 ± 3.4	40.8 ± 3.9	32.3 ± 5.4	34.0 ± 3.8	20.3 ± 2.1	16.9 ± 1.9
VCO_2 (L/min)_	0.6 ± 0.1	4.7 ± 0.5	3.7 ± 0.2	2.5 ± 0.3	3.7 ± 0.5	2.5 ± 0.4	1.9 ± 0.3
RER	0.90 ± 0.1	1.19 ± 0.1	1.01 ± 0.0	0.89 ± 0.0	1.20 ± 0.1	1.34 ± 0.1	1.24 ± 0.1
VE/VCO_2_	37.8 ± 3.8	32.6 ± 2.8	29.6 ± 2.3	28.6 ± 2.4	30.2 ± 3.3	33.4 ± 2.8	35.8 ± 2.9
O_2 pulse (mL/bpm)_	6.9 ± 2.0	21.5 ± 2.2	21.7 ± 2.0	20.1 ± 2.3	18.4 ± 2.5	13.2 ± 1.9	12.2 ± 1.4
HR _(bpm/min)_	84 ± 2.0	183 ± 8.3	168 ± 8.2	144 ± 13.7	166 ± 14.5	140 ± 1.9	126 ± 12.5
VE/VO_2_	33.9 ± 4.9	38.8 ± 3.9	29.8 ± 2.6	25.3 ± 2.2	36.3 ± 4.6	44.7 ± 4.6	44.5 ± 5.0
Speed _(km/h)_	0	16.7 ± 1.3	13.3 ± 1.6	10.1 ± 1.6	3.5	3.5	3.5

Legend-Abbreviation: CPET—Cardiopulmonary exercise testing; Rest—Rest values; Peak—Peak values at end of the test; VT2—Second ventilatory threshold; VT1—First ventilatory threshold; Reco1—First minute of recovery; Reco2—Second minute of recovery; Reco3—Third minute of recovery; WE _(W)_—work rate in watts; VE _(L)_—Ventilation in liters; VO_2 (mL/min/kg)_—Oxygen uptake in milliliters per kilogram per minute; VCO_2 (L/min)_ Carbon dioxide production liters per minute; R—Respiratory exchange ratio; VE/VCO_2_—Ventilatory equivalent for carbon dioxide production; O_2 pulse (mL/bpm)_—Oxygen pulse – oxygen uptake milliliters per heart beat; HR _(bpm/min)_—Heart Rate beats per minute; VE/VO_2_—Ventilatory equivalent for oxygen uptake.

## Data Availability

The dataset used and/or analyzed during the current study are available from the corresponding author in response to a reasonable request. Due to patient’s data, privacy data are not made available publicly.
